# Editorial: Rising stars in systems neuroscience: 2022

**DOI:** 10.3389/fnsys.2024.1414351

**Published:** 2024-05-14

**Authors:** Mazyar Fallah, Juhee Haam, Ada Ledonne

**Affiliations:** ^1^Visual Perception and Attention Lab, College of Biological Science, University of Guelph, Guelph, ON, Canada; ^2^Department of Biological Sciences, College of Science, Louisiana State University, Baton Rouge, LA, United States; ^3^Department of Systems Medicine, University of Rome Tor Vergata, Rome, Italy; ^4^Department of Experimental Neuroscience, IRCCS Santa Lucia Foundation, Rome, Italy

**Keywords:** vision, neuroinformatics, cellular neuroscience, neurophysiology, sensorimotor integration, computational modeling, AMPA, anesthesia

When a problem remains unsolved, sometimes it requires a new perspective. Often, these fresh approaches come from those early in their careers: trying new things or taking ideas and methodologies from one field of research to advance another. By doing so, new answers arise, which generate the next set of questions and make them Rising Stars. This Research Topic invited early career researchers nominated by established editors to share their latest advances across the range of systems neuroscience research areas and five Rising Stars answered the call.

We present to you three research articles, one review, and one hypothesis and theory paper. These five cover a broad span of the field. Three articles focus on visual neuroscience, one on neuroinformatics, and one on consciousness or, more precisely, its loss through anesthesia. The techniques covered span slice-based molecular and cellular neuroscience, non-human primate neurophysiology, human perception and sensorimotor integration, and computational modeling. In addition, all of them take a new look at the old phenomena.

Starting in the realm of visual neuroscience, there is a comprehensive new look at what happens when you blink. In much of active behaving vision research, participants (be they humans or non-human primates) are asked to fixate on a central point to keep their eyes still in some paradigms (with manual responses) or move their eyes around the scene in others (da Silva Lima and Ventura, 2023). In the first case, eye blinks would generally cause the trial to be aborted because visual processing was interrupted. In the latter, eye blinks are often cut out of the data to be analyzed because eye tracking was interrupted. In both cases, the paradigms generally focus on understanding visual perception without blinking, which is not how we naturally view our environment. Willett et al. review how eye blinks affect visual perception, the neural circuitry involved, and potential mechanisms that give rise to these effects while allowing us to maintain a stable perception of the world around us. Incorporating visual perception changes around eye blinks will be important to further advance our understanding of natural vision.

In a related area, saccadic eye movements (rapid, ballistic movements of the eyes that change the point of fixation) are well-studied, both from using them as a direct spatial response and as a way to measure sensorimotor integration, target selection, salience maps, and decision-making processes (Jay and Sparks, 1987; Moore and Fallah, 2001; Goldberg et al., 2006; Van der Stigchel and Theeuwes, 2006; Giuricich et al., 2023). Kehoe and Fallah turn this around through recent spatiotemporal analyses of shifts in the trajectory of saccades to a target in the presence of a distractor to hypothesize that it is competition within the most relevant stage of the *visual hierarchy* (Felleman and Van Essen, 1991) that provides the weighting of saccade motor plans that produce the trajectory shifts. Kehoe and Fallah also suggest how this method can be used to infer the timing of visual processing at different stages and how that could be used for clinical studies, enhancing the use of eye tracking as a clinical diagnostic tool.

Active vision, the combination of fixations and saccades, has been well-tested in visual search paradigms (Wolfe, 2020). Much of this research has resulted in attentional or salience models (e.g., Itti et al., 1998; Bruce and Tsotsos, 2005) that can be used to predict fixation locations when looking around a natural scene. These models tend to be accurate for predicting the first couple of fixations from stimulus-driven salience but have more trouble with exhaustive search. Later models focused on probabilistic mechanisms, which improved performance. In their work, Bujia et al. take the probabilistic approach further by employing a Bayesian approach for generating saliency maps. They showed the value in this Bayesian approach by accurately predicting the path of fixations taken until the target is found, outperforming other models in the process. Their results further advance the tenets behind Bayesian perception.

In the brain, the populational activities of multiple neurons represent complex neural information, which mediate a variety of cognitive functions (Buzsaki and Draguhn, 2004). The investigation of populational neural activities of specific brain regions and network dynamics can be performed using extracellular electrophysiological techniques, such as local field potential recordings (Hong and Lieber, 2019). Extracting information from neural recording data using the Fourier transform-based spectral analysis has been widely used to infer a change in neural oscillations by observing a change in the power of a frequency band. Using simulations, Perrenoud and Cardin further dive into this approach and demonstrate how the degree of periodicity (repetition in time) in neural signals determines the interpretation of spectral analysis results. Perrenoud and Cardin propose an alternative way of analyzing non-periodic neural data as a train of recurring events (Perrenoud and Cardin). This new framework would contribute to our understanding of complex neural data.

Anesthetics induce loss of consciousness, which is crucial for reducing sensation and pain during medical procedures. Outside the clinical setting, anesthetics have also significantly contributed to our understanding of consciousness and brain function. Ketamine is a commonly used anesthetic for induction and maintenance of anesthesia and harbors unique characteristics of enhancing neural activities in the thalamus and cortex. Until now, ketamine's actions have been explained by its antagonistic effects on the glutamatergic NMDA receptor, causing neural circuit disinhibition (Homayoun and Moghaddam, 2007; Seamans, 2008). Bieber et al. present new evidence that ketamine also modulates synaptic inputs mediated by the AMPA receptor, a different glutamate receptor subtype. Precisely, by patch-clamp recordings in brain slices of mice, Bieber et al. demonstrated that s-ketamine enhances thalamocortical and intracortical AMPA receptor-mediated synaptic transmission without altering the intrinsic properties of cortical and thalamic cells. This evidence provides interesting insights into the multifaceted cellular mechanisms behind ketamine's actions.

Taken together, these articles highlight the value of new viewpoints and new approaches by Rising Stars. It will be interesting to see how these ideas evolve within their fields over time and what the Rising Stars do next.

## Author contributions

MF: Writing—original draft, Writing—review & editing. JH: Writing—original draft, Writing—review & editing. AL: Writing—original draft, Writing—review & editing.

## References

[B1] Bruce N. D. B. Tsotsos J. K. (2005). “Saliency based on information maximization” in Proceedings of the 18th International Conference on Neural Information Process Systems, eds. Y. Weiss, B. Scholkopf, and J.C. Platt (Cambridge, MA: MIT Press), 155–162.

[B2] Buzsaki G. Draguhn A. (2004). Neuronal oscillations in cortical networks. Science 304, 1926–1929. 10.1126/science.1099745 15218136

[B3] da Silva Lima D. Ventura D. F. (2023). A review of experimental task design in psychophysical eye tracking research. Front. Hum. Neurosci. 17:1112769. 10.3389/fnhum.2023.1112769 37662635 PMC10469886

[B4] Felleman D. J. Van Essen D. C. (1991). Distributed hierarchical processing in the primate cerebral cortex. Cereb. Cortex 1, 1–47. 10.1093/cercor/1.1.1 1822724

[B5] Giuricich C. Green R. J. Jordan H. Fallah M. (2023). Target-distractor competition modulates saccade trajectories in space and object space. eNeuro 10:6. 10.1523/ENEURO.0450-22.2023 37263792 PMC10275402

[B6] Goldberg M. E. Bisley J. W. Powell K. D. Gottlieb J. (2006). Saccades, salience and attention: the role of the lateral intraparietal area in visual behavior. Prog. Brain Res. 155, 157–175. 10.1016/S0079-6123(06)55010-1 17027387 PMC3615538

[B7] Homayoun H. Moghaddam B. (2007). NMDA receptor hypofunction produces opposite effects on prefrontal cortex interneurons and pyramidal neurons. J. Neurosci. 27, 11496–11500. 10.1523/JNEUROSCI.2213-07.2007 17959792 PMC2954603

[B8] Hong G. Lieber C. M. (2019). Novel electrode technologies for neural recordings. Nat. Rev. Neurosci. 20, 330–345. 10.1038/s41583-019-0140-6 30833706 PMC6531316

[B9] Itti L. Koch C. Niebur E. (1998). A model of saliency-based visual attention for rapid scene analysis. IEEE Trans. Patt. Anal. Mach. Intell. 20, 1254–1259. 10.1109/34.73055817688904

[B10] Jay M. F. Sparks D. L. (1987). Sensorimotor integration in the primate superior colliculus. I. Motor convergence. J. Neurophys. 27, 22–34. 10.1152/jn.1987.57.1.22 3559673

[B11] Moore T. Fallah M. (2001). Control of eye movements and spatial attention. PNAS 98, 1273–1276. 10.1073/pnas.98.3.1273 11158629 PMC14744

[B12] Seamans J. (2008). Losing inhibition with ketamine. Nat. Chem. Biol. 4, 91–93. 10.1038/nchembio0208-91 18202677

[B13] Van der Stigchel S. Theeuwes J. (2006). Our eyes deviate away from a location where a distractor is expected to appear. Exp. Brain Res. 169, 338–349. 10.1007/s00221-005-0147-2 16273397

[B14] Wolfe J. M. (2020). Visual search: how do we find what we are looking for? Ann. Rev. Vis. Sci. 5, 539–562. 10.1146/annurev-vision-091718-015048 32320631 PMC13537705

